# Plant Macrofossils Reveal Aquatic Macrophyte Successions of a Typical Shallow Lake (Huanggai Lake, China) in the Past Century

**DOI:** 10.3390/plants11111413

**Published:** 2022-05-26

**Authors:** Qijuan Cheng, Liangfang Li, Xuhui Dong, Yan Li, Giri Kattel

**Affiliations:** 1 School of Geography and Remote Sensing, Guangzhou University, Guangzhou 510006, China; 2112001032@e.gzhu.edu.cn (Q.C.); 2112101029@e.gzhu.edu.cn (L.L.); 2 Centre for Climate and Environmental Changes, Guangzhou University, Guangzhou 510006, China; 3 School of Geographical Sciences, Nanjing University of Information Science and Technology, Nanjing 210044, China; gkattel@unimelb.edu.au; 4 Department of Infrastructure Engineering, University of Melbourne, Melbourne 3010, Australia; 5 Department of Hydraulic Engineering, Tsinghua University, Beijing 100084, China

**Keywords:** plant macrofossil, aquatic macrophyte, Huanggai Lake, Yangtze River Basin, lake evolution

## Abstract

Aquatic macrophytes are one of the important biotic components of shallow lake ecosystems. Understanding the long-term evolution of the macrophyte community is crucial for lake management. Huanggai Lake, a typical shallow lake in the middle reach of the Yangtze River, was selected as the research site for this study. Based on ^210^Pb/^137^Cs dating, aquatic plant macrofossils were used to reconstruct the succession of aquatic macrophytes in the past century. Our results show that the lake maintained a consistent natural state before 1940, with a relatively low abundance of aquatic plants dominated by species such as *Najas minor*. From 1940 to 1974, human activities gradually intensified in the lake leading to the emergence of eutrophic species such as *Potamogeton maackianus*, along with the increasing abundance of other emergent and floating aquatic macrophytes. Since 1974, more pollution-resistant, emergent species such as *Potamogeton maackianus* and *Potamogeton crispus* have become dominant. The abundance of aquatic macrophytes reached its maximum in the early 1990s. Combined with macrofossil succession and other multiple sedimentary proxy analyses, driving mechanisms for aquatic macrophytes are discussed. Both the nearby Liangzi Lake and Huanggai Lake share many common features of aquatic plant evolution. This study is the first of its kind to use plant macrofossils (with identifiable images) as a proxy for aquatic macrophyte succession in a shallow Yangtze lake. In absence of long-term monitoring records, this study highlights the increased application of plant macrofossils for reconstructing the vegetation dynamics and restoration of degraded lakes exposed to severe anthropogenic impacts over the past century.

## 1. Introduction

Increasing human activities due to population growth have brought unprecedented disturbances to the Earth’s surface, and aquatic ecosystems, which are generally facing serious environmental degradation, loss of biodiversity, and degradation of ecosystem services worldwide [1]. One of the most prominent features observed in many shallow lakes around the world is bistability, a condition of the disappearance of the aquatic macrophyte community, along with accelerated environmental degradation [2,3]. Many shallow lakes, including the lakes within the Yangtze River Basin, have shifted from “clear water” dominated by macrophytes to “turbid water” dominated by algae, resulting in the loss of key ecosystem services, including fish biomass and clean water supply for human consumption. The condition of reduced water quality has seriously restricted overall socio-economic development in the basin [4,5]. Understanding the succession of the lacustrine community in the basin is a prerequisite for preventing human-induced ecological catastrophes through timely restoration and management [6]. However, due to the limited availability of historical records, such information is often scarce, and the ecological investigation based on contemporary records in lakes is insufficient to provide unbiased outcomes on species succession and characteristics of the aquatic macrophyte community under different environmental conditions in the past [7].

Seeds and plant residues produced by various terrestrial and aquatic higher plants are scattered in sediments and preserved as plant macrofossils [8,9]. Compared with pollens, plant macrofossils are relatively heavier in density and are not easily diffused. Therefore, with the aid of pollens, the past growth of macrophytes can more accurately be evaluated in lowland shallow floodplain lakes and coastal estuaries [9]. The plant macrofossils are also easier to identify at the species level, further refining the plant community types and becoming increasingly ideal for past aquatic macrophyte community reconstruction [10]. There are several case studies using plant macrofossils in lake sediment to understand the historical succession of aquatic macrophytes in the United States and Europe [9,10]. For example, Madgwick et al. [11] reconstructed the information of aquatic macrophytes in Barton broad, East England, in the past 200 years, through the identification of aquatic plant macrofossils in lake sediments and provided corresponding management measures of the lake based on the plant macrofossil stratigraphy.

Aquatic plants exhibit significant features in the biogeographic distribution, along with their geographic preferences and the background of the geographic location such as the climatic and nutritional status of soil and sediment. For example, unlike deep plateau lakes, shallow lakes in lowland floodplain areas have abundant aquatic plants due to the shallowness and potential high concentration nutrient background [12,13,14]. Studies on the evolution of plant macrofossils in sediments, such as the taxa and content of seed banks, have become potentially useful to determine the dredging depth of sediment and provided important information for the conservation of rare species in some regions [15,16,17]. For example, Sayer et al. [17] reestablished former charophyte populations through the germination of dormant oospores. They have recommended the removal of different sediment layers from the three geographically distributed lake systems—Cromes North, Upton Little, and Little Broads—so that rich sediment layers with oospores would be exposed for germination. Bishop et al. [16] used macrofossils as a proxy for the succession of rare macrophyte species, including *Najas flexilis*, in a Scottish pond; in conjunction with information on the dispersal potential, the study revealed an apparent decline in the abundance of *Najas flexilis* in Scotland over the past 100 years. This kind of study has become useful for providing biogeographical distribution of rare species, including *Najas flexilis*, in the past and is also useful to lake conservation programs in Scotland today. Given its roles in socio-economic development, extensive research on plant macrofossils (including seed banks) at multiregional and longer time scales is becoming increasingly significant for basin-wide aquatic macrophyte restoration and biodiversity protection programs in the Yangtze River Basin.

Many shallow lakes in the middle and lower reaches of the Yangtze River Basin favor the growth of aquatic macrophytes, while they have been seriously exposed to severe environmental stressors in recent decades [18]. The increased input of nutrients, the change in fluvial systems and hydrology, the intensified human activities such as urbanization and industrial development, and the rapid rates of climate and environmental changes all have been the major drivers of the succession of aquatic macrophytes in the middle and lower reaches of the Yangtze River Basin [19,20,21]. However, there are only a few relevant reports available on the evolutionary trend and causal mechanisms of macrophyte succession in the basin [22]. This study chose the Huanggai Lake, a typical shallow lake in the middle reach of the Yangtze River Basin, to explore the century-scale macrophyte succession. At present, Huanggai Lake contains rich aquatic macrophytes, potentially serving as an ideal site for preserving macrofossils over time. As no monitoring records of aquatic vegetation are available on Huanggai Lake to date, we aimed to reconstruct the history of local aquatic macrophytes, provide new information regarding temporal changes in the aquatic macrophytes, and facilitate ecological restoration and conservation in Huanggai Lake and other similar lakes. We hypothesized that the nutrient changes in the lake have triggered a transition from oligo mesotrophic submerged-flora-dominated condition to the dominance of submerged eutrophic species. Additionally, we expect a common evolutional pattern in similar shallow lakes (i.e., Liangzi Lake), given their similar geographic background and lake conditions. To test these hypotheses, we reconstructed the long-term evolution process of macrophytes using multiproxy paleolimnological (plant macrofossil, pollen, magnetic susceptibility, and geochemistry) and documentary records, to identify drivers that could affect the macrophyte dynamics.

## 2. Materials and Methods

### 2.1. Study Site

**Figure 1:**
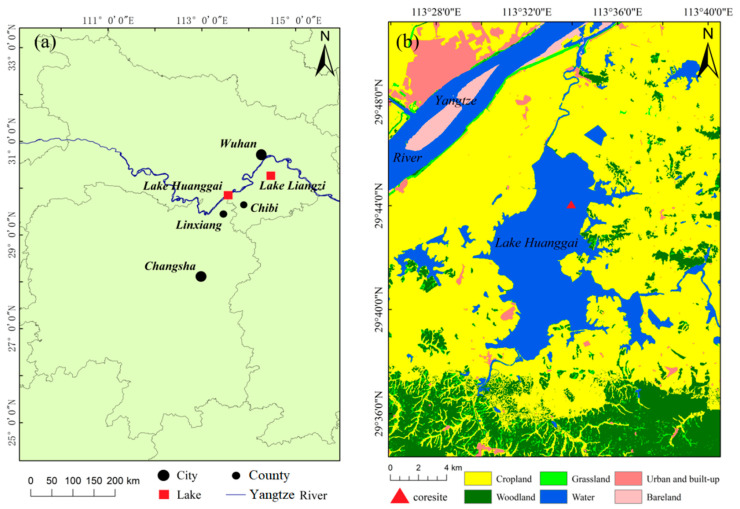
Geographical location of Huanggai Lake with (**a**) the map exhibiting two lakes, Huanggai Lake and Liangzi Lake, along the Yangtze River; (**b**) the land use of Huanggai Lake Basin.

Huanggai Lake (113°29′48″–113°36′40″ E, 29°37′00″–29°46′12″ N) crosses Linxiang City, Hunan Province, and Chibi City, Hubei Province (Figure 1). Nearly two-thirds of the western and southern banks are under the jurisdiction of Linxiang City [23]. Huanggai Lake is close to the southern bank of the middle reaches of the Yangtze River and belongs to the Dongting Catchment, with a water area of 86 km^2^. The maximum water depth is 6.3 m, with an average water depth of 5.6 m. The water storage capacity is 3.93 × 10^8^ m^3^, with a catchment area of 1677 km^2^ and a recharge coefficient of 19.5 [24]. The lake water is mainly supplied by precipitation and catchment surface runoff. The water from the Xindian River, Yuantan River, and areas around the lake is regulated and stored by Huanggai Lake and discharged northward from Yapengkou River into the Yangtze River. The area belongs to the subtropical monsoon climate, with four distinct seasons and the same period of rain and heat. The average annual temperature is 17 °C, and the average annual precipitation is 1582.5 mm.

The major types of land use in the catchment are agriculture, forestry, paddies, and urban settlements (Figure 1). The past 100 years, particularly the recent 30 years, have seen rapid growth of the local population and fast economic development in the study region, causing increased soil erosion in the catchment. Intensive human activities impacted local water quality. According to the Xianning water resources bulletin and our survey [23,24], the overall water quality of Huanggai Lake is currently poor (Table 1). The seasonal total phosphorus (TP) and total nitrogen (TN) ranged between 0.09–0.17 and 1.5–3.5 mg/L, respectively. According to our field survey in 2019, most parts of the lake was covered by abundant aquatic macrophyte, including *Hydrilla verticillata*, *Ceratophyllum demersum*, *Vallisneria natans*, and *Potamogeton* sp., and the main types of plant formations are *Euryale ferox* community and *Nymphoides* community.

### 2.2. Documentary Data on Local Social and Environmental Background

**Figure 2:**
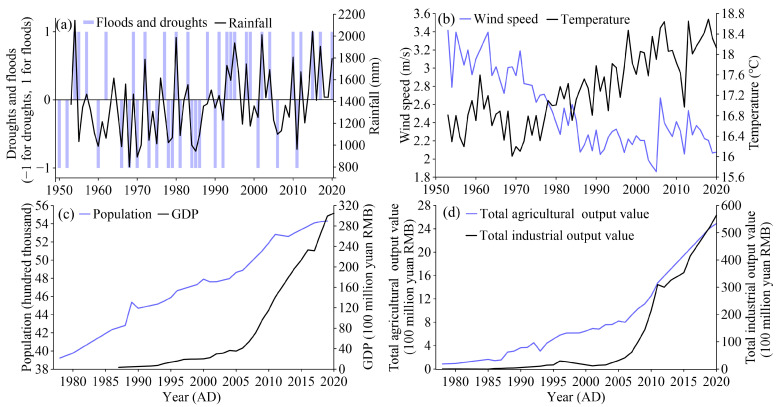
Historical records for the Huanggai Lake Basin. The records include (**a**) rainfall, flood, and drought data; (**b**) temperature and wind speed data; (**c**) population and GDP data; (**d**) total agricultural output value and total industrial output value.

The population, Gross Domestic Product (GDP), total agricultural output value, and total industrial output value of Linxiang city, near Huanggai Lake, were collected to reflect the social and economic development of the basin. The data are available from the Linxiang almanac and China’s social and economic big data research platform (https://data.cnki.net/, accessed on 14 January 2022). The meteorological data were collected from the network of China Meteorological Science Data Center (http://data.cma.cn/, accessed on 14 January 2022), and the data of annual temperature, annual precipitation, and annual wind speed of Yueyang station, which is 60 km away from the Huanggai Lake basin, were selected to represent the change in the climate of the basin. The data on natural disasters such as floods and drought periods are from the Linxiang almanac [25]. In Figure 2, the historical records of the specific year of flood and drought disasters are presented, in which a flood year is recorded as 1, a year of drought is recorded as −1, and ones without natural disasters are recorded as 0. The amount of precipitation is generally consistent with the frequency of flood and drought disasters (Figure 2). The average temperature fluctuated upward, and the wind speed decreased. The population has shown an obvious upward trend since 1978, and the GDP has increased rapidly since 2007. The total agricultural output value and total industrial output value both show increasing trends.

### 2.3. Sediment Sampling and Analyses

In July 2019, a sediment core (HG5) was collected using a gravity corer at a water depth of 5.9 m on the northern bank of Huanggai Lake (Figure 1). Contiguous subsamples were sliced at 1 cm intervals in the field and stored at 4 °C in the laboratory until further processing. Laboratory analysis was subjected to ^210^Pb and ^137^Cs, magnetic susceptibility, element, plant macrofossils, and pollens. Levels of ^210^Pb and ^137^Cs activity were measured via direct gamma spectrometry using a well-type, coaxial, low background, and intrinsic germanium detector (HPGe GWL-120-15). ^137^Cs activity was used to identify the 1963 nuclear weapons peak. ^210^Pb chronologies were calculated using the constant rate of supply (CRS) model and were verified using the highest ^137^Cs activity in 1963 [26].

Samples for magnetic susceptibility measurements at 2 cm intervals were air-dried below 40 °C, packed in non-magnetic plastic cube boxes (2 cm × 2 cm × 2 cm), and weighed on an electronic balance with a precision of 0.01 mg. Low- (976 Hz) and high-frequency (15,616 Hz) MS (mass-specific χ_lf_ and χ_hf_, respectively) were measured using a Kappa-bridge MFK1-FA (AGICO). Frequency-dependent magnetic susceptibility was calculated from the expression χ_fd_ (%) = [(χ_lf_ − χ_hf_)/χ_lf_] × 100. To analyze the geochemical elements, the samples (2 cm interval) were air-dried at room temperature and then ground and sealed using 100 µm mesh. A series of elements were analyzed, including P, Pb, and Cu, by applying inductively coupled plasma mass spectrometry (ICP-MS).

A volume of 50 cm^3^ sediment for each sample was prepared using the standard methods for plant macrofossil analysis [9]. Samples were washed through a 250 μm mesh sieve, and the residue was examined under a stereomicroscope at 10–100× magnification. A subsample, approximately a quarter of the total sample, was subsequently washed through a 125 μm sieve and analyzed at higher magnification, for determining smaller remains. The content of plant macrofossils is expressed by the content of the sample per unit volume (no./100 cm^3^). The identification of plant macrofossils mainly includes seeds, leaves, and fruits, most of which can be identified at the species level. In the procedure of counting the macrofossils, a whole individual is regarded as one, and a half is regarded as incomplete. The identification follows the published species plate literature [8,27,28], as well as the Chinese plant species information database (http://db.kib.ac.cn/, accessed on 4 December 2021). The samples for pollen analysis were processed through a standard procedure [29]. A minimum of 500 pollen grains was counted from every sample. Lycopodium tablets were added to each pollen sample as a tracer for determining the pollen concentration.

### 2.4. Statistical Methods

All statistical analyses of diatom assemblages were based on percentage abundances and included 13 plant taxa, with >2% abundance in at least one sample. Zones in the core were identified using the constrained incremental sum of squares (CONISS) method in TILIA and TILIAGRAPH computer programs [30]. To extract the major pattern of aquatic plant communities, a principal component analysis (PCA) was applied to macrofossil data and the scores of the first axis (PC1) were extracted and considered indicative of the major changing trend. The species percentage data were squared-root-transformed, and rare taxa were downweighed prior to the analysis.

## 3. Results

### 3.1. Chronology

**Figure 3:**
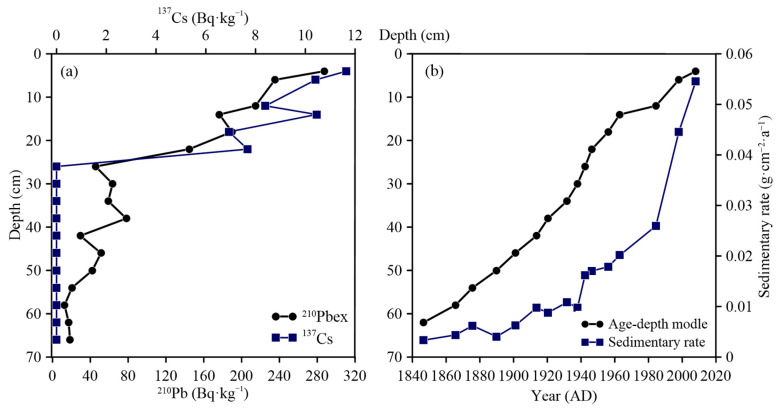
Dating results of the core from Huanggai Lake: (**a**) downcore variations in ^137^Cs and ^210^Pb_ex_ activities; (**b**) age–depth model and sedimentary rates.

The dating of Huanggai Lake sediment was mainly based on ^210^Pb and ^137^Cs radioisotopes. Considering potential disturbance in the catchment and compaction, sedimentation rates were not constant, and the chronology was, therefore, estimated using the constant rate of supply (CRS) model [26]. The ^210^Pb_ex_ activity in the sediment core showed an exponential declining trend (Figure 3). The variation characteristics of ^210^Pbex and ^137^Cs specific activities in the sedimentary column with depth are shown in Figure 3a, while the corresponding relationship between the age and depth of the sedimentary column is shown in Figure 3b; the age corresponding to the depth of 62 cm is about 1856. Sediment accumulation rates kept increasing over the past 180 years (Figure 3b), with two significant increments: one in the 1940s (<0.01 g/cm^2^·a) and the other in the1980s (>0.02 g/cm^2^·a).

### 3.2. Macrophyte Flora

**Figure 4:**
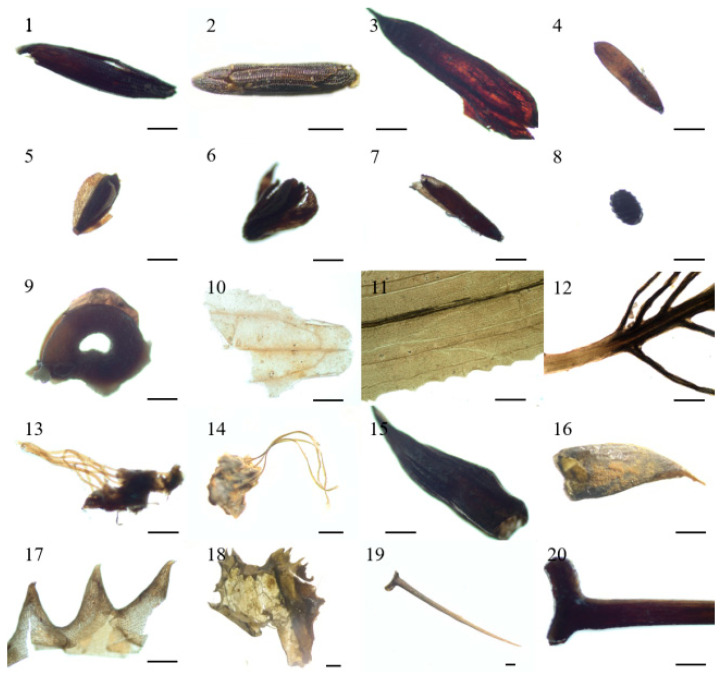
Images of dominated plant macrofossils in Huanggai Lake, with each unit scale as 0.5 mm. 1–2: *Najas minor* seed; 3: *Hydrilla verticillata* seed; 4: *Vallisneria natans* seed; 5–7: *Vallisneria denseserrulata* seed; 8: *Chara* sp. oospore; 9: *Potamogeton* sp. seed; 10–11: *Potamogeton maackianus* leave; 12: *Myriophyllum spicatum* leave; 13–14: *Euryale ferox* leave; 15–16: *Potamogeton crispus* seed hood; 17–18: *Potamogeton crispus* turion teeth; 19–20: *Ceratophyllum demersum* seed spine.

A total of 11 species of aquatic plants were identified in the HG5 core, including submerged and floating plant species, respectively (Figure 4). Submerged plants were the most common macrophytes, which include *Najas minor*, *Hydrilla verticillata*, *Vallisneria natans*, *Vallisneria denseserrulata*, *Chara* sp., *Potamogeton* sp., *Potamogeton maackianus*, *Myriophyllum spicatum*, *Ceratophyllum demersum*, *Potamogeton crispus* in the lake. Floating plants were recorded as relatively less common, compared with submerged macrophytes, which include *Euryale ferox*.

**Figure 5:**
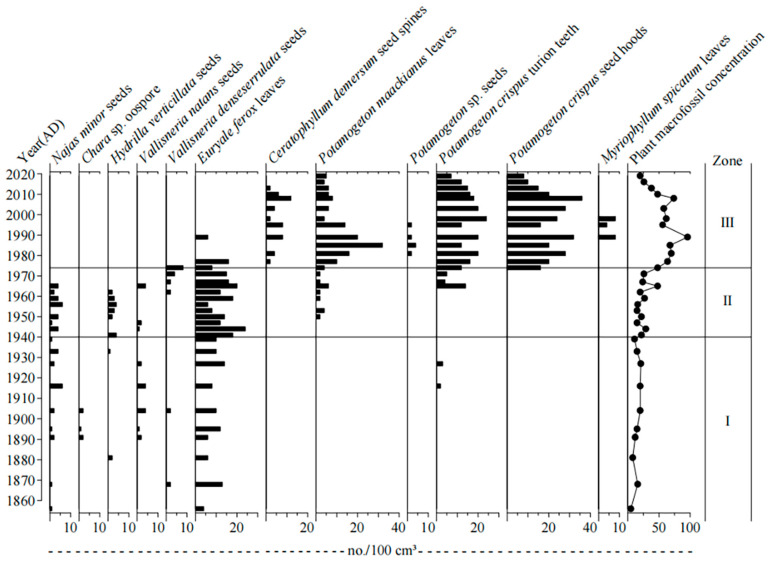
Vertical changes in aquatic plant macrofossils in the Huanggai Lake core.

The changing trend of the main aquatic plant macrofossils is shown in Figure 5.

Zone I (AD 1856–1940): There were abundant species of aquatic macrophytes recorded in this zone, with the most common submerged macrophytes being *Najas minor*, *Hydrilla verticillata*, *Vallisneria natans*, *Vallisneria denseserrulata*, and *Chara* sp., respectively. The dominant species of the plant community present in this zone were *Najas minor* and *Vallisneria natans*, respectively. The absolute concentration of plant macrofossils was relatively at a low level, as the number of aquatic macrophytes was recorded as small; in addition, the general macrophyte coverage was low.

Zone II (AD 1940–1974): In this zone, the aquatic macrophyte was still dominated by *Najas minor*, *Hydrilla verticillate*; however, *Chara* sp. suddenly disappeared, the number of *Euryale ferox* primarily began to decrease and then increased, and *Potamogeton maackianus* and *Potamogeton crispus* both began to emerge in this zone. In general, the concentration of plant macrofossils began to increase, and the abundance of aquatic macrophytes increased.

Zone III (AD 1974–2019): In this zone, the number of *Najas minor*, *Hydrilla verticillata*, *Vallisneria natans*, *Vallisneria denseserrulata*, and *Euryale ferox* remained decreased or even disappeared. *Potamogeton crispus* and *Potamogeton maackianus* became dominant species, and *Ceratophyllum demersum* was regularly present. The concentration of plant macrofossils increased significantly, reaching the maximum density in about 1989, and then fluctuated and decreased. In this zone, the number of aquatic macrophytes was relatively large, and the macrophyte coverage was also high.

### 3.3. Ordination of Macrofossil Records

**Figure 6:**
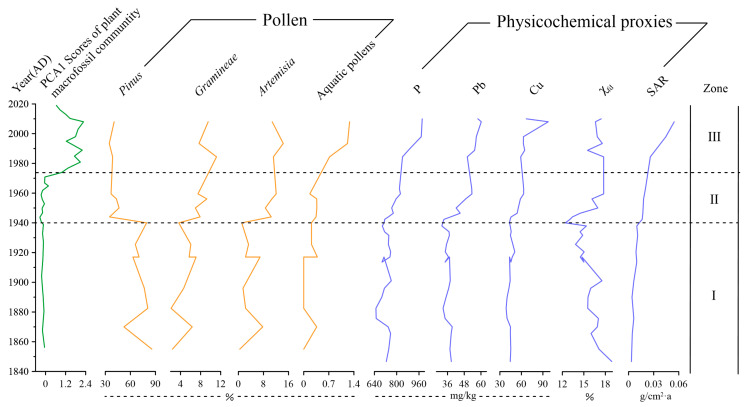
Stratigraphic changes in sedimentary proxies, including pollen, geochemical, magnetic, and sediment rates in the Huanggai Lake. The zonation followed the one revealed by macrofossil records.

The first principal component (PC1) of plant residue (both genera and species) samples explained 40.8% of the total variance. Figure 6 shows the comparison of the comprehensive analysis results of PC1 scores, the percentage content of *Pinus*, *Gramineae*, *Artemisia,* aquatic pollens P, Pb, Cu, and χ_fd_, as well as the sedimentation rate (SAR) of plant macrofossils samples. The PC1 score of plant macrofossils did not show a significant change before 1974 but fluctuated and increased after 1974 and began to decline in 2008. PC1 scores of plant macrofossil community changes were observed as more obvious in 1966, 1994, and 2010, respectively. *Pinus* generally showed a downward trend before 1940 and decreased rapidly after 1940; *Gramineae* and *Artemisia* showed a fluctuating upward trend, which was the most obvious after 1940; Aquatic pollens remained basically stable before 1940 and increased significantly after 1940, especially after 1970; P, Pb, and Cu changed little before 1940 but fluctuated and increased greatly after 1940, and Cu reached the highest value in 2008; χ_fd_ showed a downward trend before 1940 and increased thereafter. SAR showed a significant upward trend, especially after 40 years and again after 80 years.

### 3.4. Multiple Sedimentary Proxy Analysis

Multiple proxies were conducted to reveal the environmental change in the catchment (Figure 6).

Zone I (AD 1840–1940): PC1 scores of macrofossils were relatively stable. The pollen and physicochemical proxies (*Gramineae*, *Artemisia*, aquatic pollens, P, Pb, Cu, SAR) remained relatively stable. Pollen was dominated by *Pinus*, with an average abundance of 83%. Elemental phosphorus, Pb, and Cu were found in low concentrations. Sediment accumulation rates were of low values (<0.02 g/cm^2^·a) but exhibited a small increasing trend.

Zone II (AD 1940–1974): PC1 scores of macrofossils gradually increased. This zone was characterized by a fluctuant increase in Pb, P, Cu, SAR, χ_fd_, *Gramineae*, *Artemisia*, and aquatic pollens. *Pinus* decreased suddenly to 40%.

Zone III (AD 1974–2019): This zone was characterized by a high PC1 score of plant macrofossil and a continuous increase in aquatic pollens, P, and SAR. Cu and Pb displayed a slight increase, and the *Gramineae*, *Artemisia*, and χ_fd_ had a slight drop, while *Pinus* did not show a marked change.

## 4. Discussion

### 4.1. Ecological Habits of Aquatic Macrophytes in Huanggai Lake

Over the past hundred years, the succession of aquatic macrophytes in Huanggai Lake is closely related to the dynamics of nutrient inputs. The mixture of two major nutrients—P and N—into the lake water was minimal in the past. The aquatic plant community has changed from species requiring a relatively nutrient-poor water body, with shorter growth and a preference for clear water, such as *Vallisneria natans*, *Najas minor*, etc., to those needing a nutrient-rich water body, with taller growth and pollution resistance, such as *Potamogeton crispus*, *Ceratophyllum demersum*, etc. The concentration of plant macrofossils remained relatively at a low level before 1940 and began to rise afterward. Plant macrofossils increased rapidly particularly after 1970, revealing an improvement in aquatic macrophyte coverage in the lake.

**Figure 7:**
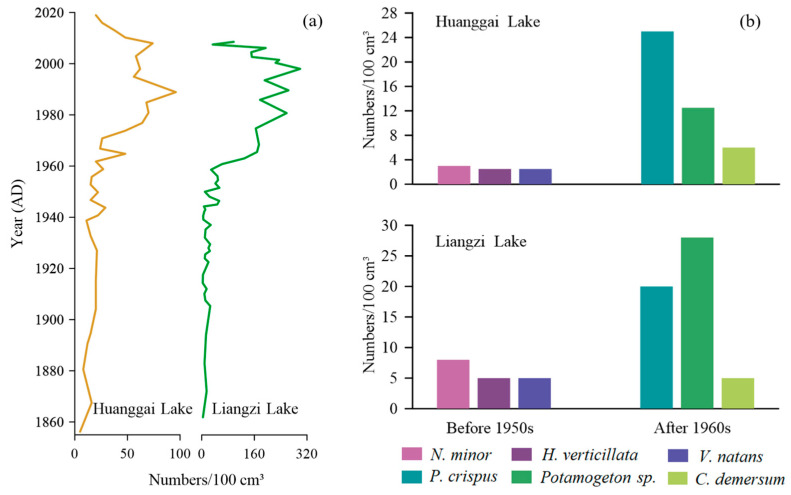
Comparison of plant macrofossil characteristics between Huanggai Lake and Liangzi Lake in different periods: (**a**) temporal trend of total concentration; (**b**) average concentration of main aquatic plant species in two different periods (natural condition before 1950 and stronger human impact after 1960s).

*Najas minor*, which prefers good water quality with poor nutrition, is sensitive to the degrading environment (see Table 2). This species was mostly recorded in sediments prior to 1940. *Chara* sp. and *Hydrilla verticillata*, which prefer similar ecological habitats of *Najas minor* (Table 2), were also recorded in the Huanggai lake sediment prior to 1940, reflecting a preference for low nutritional level of the lake [31]. There are three types of *Vallisneria* in China—*Vallisneria natans*, *Vallisneria* *denseserrulata,* and *Vallisneria spinulosa*—which are widely distributed in lakes in the middle and lower reaches of the Yangtze River [32]. *Vallisneria natans* has a strong adsorption capacity for pollutants and is the main submerged plant to reduce water pollution and alleviate lake eutrophication [33]. In our study, as well as in recent investigations by other teams, *Potamogeton maackianus* is recorded as dominant in Huanggai Lake (Figure 5 and Figure 7). They are commonly recorded in many mesotrophic lakes. It is also the main constructive species (species of having water purification capacity) of lakes in the middle and lower reaches of the Yangtze River, as this species plays an important role in maintaining the clear states of the lakes in the region [34]. *Potamogeton crispus* was recorded as dominant in the sediments of Huanggai Lake. This species is a pollution-resistant species growing in meso-to-eutrophic lakes, and it also has strong adaptability and purification capacity in degraded water [35]. *Myriophyllum spicatum* and *Ceratophyllum demersum* usually prefer eutrophic water bodies (Table 2). Both *Myriophyllum spicatum* and *Ceratophyllum demersum* are indicative of lake eutrophication [36]. Studies suggest that the response of different aquatic macrophytes to nutrients is usually different, reflecting the rapid change in trophic status, lake macrophyte succession, and quantitative dynamics of the aquatic macrophyte community over time. The most common aquatic macrophytes and their characteristics based on their life history records in Huanggai Lake are shown in Table 2.

### 4.2. Succession of Aquatic Macrophyte in Huanggai Lake: Patterns and Drivers

From 1856 to 1940, Huanggai Lake had good natural connectivity and strong hydrodynamic conditions with the Yangtze River. Pollens of *Gramineae*, *Artemisia*, and the other nutritional elements such as P were at a low level. Human development intensity in the basin was also low during this period. The lake was in a low nutritional state, and the lake environment would maintain a self-organizing ecosystem, which means the lake ecosystem would regulate pollutants itself. This was possible due to the presence of a rich, submerged macrophyte community in the lake. During this time, the main submerged macrophytes—*Najas minor* and *Vallisneria natans*—preferred a nutrient-poor, clear-water environment for their growth [31]. *Potamogeton crispus*, which prefers mesotrophic water, was also present during this time but not as the dominant species of the lake. The absence of eutrophic genera and species such as *Ceratophyllum demersum* and *Myriophyllum* was indicative of fewer nutrient inputs such as P loads into the lake [38]. The nutrient-poor lake environment led to low concentrations of plant macrofossils and is consistent with low concentrations of aquatic pollens (Figure 6).

From 1940 to 1974, Huanggai Lake had superimposed traces of human interventions, with significant deviation from the natural evolution. The concentrations of *Pinus* pollens decreased during this time, indicating increased deforestation in the basin. The corresponding increase in spores and pollen of *Gramineae* and *Artemisia* indicates the strengthening of agricultural cultivation in the region [39]. The increase in precipitation and frequent floods and erosion during this time intensified catchment materials entering the lake, causing increased magnetic susceptibility and deposition rates. The foundation of the people’s Republic of China in 1949 led to the increase in aquaculture and other water resource development activities in Huanggai Lake Basin, as well as the large-scale land reclamation program that occurred around the lake. Meanwhile, the construction of small factories and inputs of heavy metals including Pb, Cu, and nutrients such as P began to increase in the basin, leading to noticeable changes in the lake environment, including a shift in the aquatic macrophyte community [40]. Although *Najas minor* and *Hydrilla verticillata* were still the main aquatic macrophytes at this period, the presence of mesotrophic water-preferring species such as *Potamogeton maackianus* and *Potamogeton crispus*, and the increase in floating leaf plant *Euryale ferox*, were indicative of a gradual increase in the lake water nutrient level [34]. The flood control levee that was built in 1958 could have led to disruptions in the hydrodynamics, including the connectivity between Huanggai Lake and the Yangtze River [23]. A stable hydrological environment of the lake would have been conducive to the expansion of certain types of aquatic macrophytes such as *Potamogeton maackianus*, *Ceratophyllum demersum*, and *Myriophyllum* *spicatum* during this period. As the stability of hydrodynamic conditions was weakened, the nutrient retention time was prolonged, and nutrient enrichment was accelerated, which consequently promoted the absorption and utilization of nutrients by aquatic organisms [41,42]. The increase in aquatic pollen concentrations during this period (Figure 5 and Figure 6) indicates that the dynamics of nutrients and hydrology were more conducive to the growth and development of submerged macrophytes [22]. In a similar research case, the continuous inputs of exogenous nutrients in nearby Longgan Lake were reported to have led to the increased sedimentary TP, with the expansion of submerged macrophytes [43].

After 1974, human activities further intensified the lake catchments, leading to lower *Pinus* pollens and higher *Gramineae* and *Artemisia* contents. Agricultural cultivation around the catchments increased with the occurrence of further deforestation and land reclamation, leading to the loss of soil and water. After the 1980s, P, N, and other chemical fertilizers were widely used in the basin [25]. Investment and use of large amounts of chemical fertilizers such as P was reported significant for the marked loss of farmland P into the lake [44]. At the same time, the growth trend of heavy metal elements Pb and Cu was found to be synchronized with that of nutrient element P, causing a significant change in the lake environment. The input of industrial and agricultural sewage was intensified by expanding urbanization, leading to eutrophication and heavy metal pollution in Huanggai Lake.

Rising temperature and decreasing wind fluctuations weakened wind currents and flows, which would have provided favorable conditions for lake eutrophication further [45]. In shallow lakes, wind-induced mixing of sediment and nutrient occurs, leading to a conducive environment for algal growth and eutrophication [46]. This evidence is also revealed by the PC1 scores of plant macrofossils (Figure 6). Macrophytes such as *Ceratophyllum demersum* and *Myriophyllum spicatum* were often found in moderately eutrophic lakes [47], while the abundance of *Potamogeton maackianus*, *Ceratophyllum demersum*, and *Myriophyllum* *spicatum* during this time was found to be increased, reflecting the very strong response of macrophytes to succession and eutrophication. Some aquatic macrophytes such as *Najas minor* and *Hydrilla verticillata* were found to be highly sensitive to environmental changes but not resistant to low light and high nutrition, while some other macrophytes were not present and even disappeared from the lake, replaced by pollution-resistant and tall aquatic macrophytes such as *Potamogeton maackianus* and *Potamogeton crispus*. Studies suggest that when nutrient concentrations exceed a certain threshold, submerged macrophytes will degrade, and floating plants and algae will grow in large numbers [48,49]. According to the records of plant macrofossils in Huanggai Lake, submerged macrophyte is still the main macrophyte community at present, but the trend of aquatic macrophyte, in general, tends to decline. Phenomenon reflecting such succession in the Huanggai lake ecosystem, as revealed by different macrophyte species, is becoming increasingly useful for managing the nutrient level in the lake water and restoring lowland riverine habitats in the Yangtze River Basin.

### 4.3. Comparison of Plant Macrofossil Records with Liangzi Lake in the Same Region

Huanggai Lake and Liangzi Lake are located about 150 km apart in the same region (Figure 1). They share similar climatic and hydrological conditions with similar compositions and succession of aquatic macrophyte communities. For instance, over the past 160 years, the aquatic macrophytes *Chara* sp., *Najas minor*, *Najas marina*, *Nitella* sp., *Hydrilla verticillata*, *Vallisneria denseserrulata*, *Potamogeton* sp., *Ceratophyllum demersum*, *Potamogeton crispus*, *Myriophyllum spicatum*, *Nymphaeaceae*, *Euryale ferox*, *Trapa natans*, *Typha* sp., *Juncus* sp., and *Gloeotrichia echinulata* have been commonly recorded in Liangzi Lake [50]. However, Liangzi Lake contains richer macrophyte diversity, with higher aquatic macrophyte genera and species than Huanggai lake. Liangzi Lake has maintained the stable state of a macrophyte-dominated lake [50]. Based on the aquatic macrophyte abundance succession in Figure 7a, prior to human interference (c. 1950), the concentrations of plant macrofossils in the two lakes were rather low. Both lakes were in a state of poor nutrients, with low levels of plant growth [50]. After the 1950s, the concentrations of plant macrofossils began to rise gradually, which we believe was due to the increase in water column nutrients influenced by human intensification [19]. The growth and development of aquatic macrophytes in both lakes were relatively high. In around the 1990s, the aquatic macrophytes began to decline [50,51]. The concentration of plant macrofossils in Liangzi Lake was recorded higher than in Huanggai Lake, which was due to the richer diversity of aquatic macrophytes in Liangzi Lake.

From the perspective of the main aquatic macrophyte genera and species present in two lakes as shown in Figure 7b, before the 1950s, *Najas minor* were the main dominant species for both lakes. There were, however, higher numbers of average *Najas minor* present in Liangzi Lake, and there was a slight difference between the numbers of *Hydrilla verticillata* and *Vallisneria natans* recorded between the two lakes. After the 1960s, *Potamogeton crispus*, *Potamogeton* sp., and *Ceratophyllum demersum* were the dominant genera and species in both lakes. However, there were some differences found in the presence of genera and species of *Potamogeton* sp. More *Potamogeton* sp. were recorded in Liangzi Lake, while more *Potamogeton crispus* species were found in Huanggai Lake (Figure 7b). While it is interesting to see these differences in macrophyte succession in two lakes with similar climatic and hydrodynamics conditions over time, what causes those successive differences in the succession is a matter of future research. Even though two lake environments are roughly the same, and also consistent with the trend of aquatic macrophyte succession showing the initial disappearance of short and slow-growing *Najas minor* and *Chara* sp. and transitioning to the taller macrophytes such as *Potamogeton maackianus*, *Ceratophyllum demersum*, and *Myriophyllum* *spicatum* with rapid rates, could be associated with microclimate and species-specific habitat preference [17,50]. A similar trend was observed in shallow wetlands in the UK. For instance, aquatic macrophytes of Felbrigg Hall Lake began with *Chara* sp. with low plants and slow growth and gradually transitioned into macrophytes dominated by fast growth and large plants such as *Potamogeton* sp. and *Ceratophyllum demersum* [17]. However, there were also differences in the timing and magnitude of plant growth in those British lakes. In Huanggai Lake, the decreasing coverage of *Najas minor*, which is sensitive to changes in nutrients, indicated that Huanggai Lake corresponded to increased total phosphorus; while in Liangzi Lake, *Najas minor* indicated a recession trend around 1985. Differences in the synchronism of the recession trend as indicated by *Najas minor* may be due to the difference in the loading of nutrient levels between the two lakes over time [50].

### 4.4. Implications for Lake Environmental Governance and Restoration

This study provides important information for the restoration of aquatic macrophytes in Huanggai Lake. Contemporary lake monitoring can provide information about aquatic macrophytes only for a shorter period; as a result, it is not possible to clearly decipher the lake evolution through species succession related to different environmental conditions in the past. In the process of plant restoration, human activity can always cause new disturbances to the lake ecosystem, which is difficult to avoid [52]. In many cases, human activity has resulted in the invasion of new species such as *Eichhornia crassipes* [53]. Information on long-term environmental changes, together with the understanding of lake evolution, has become increasingly invaluable to fixing issues such as species invasion in many parts of the world [54]. Reconstruction of plant macrofossils derived from lake sediments can potentially detect long-term lake environmental change and identify the phases of macrophyte succession over different periods in the past [9]. At a time of reconstruction, an understanding of the evolutionary history and succession of aquatic macrophytes has greater significance in the ecological restoration of shallow lakes. In many countries, including the United Kingdom, the application of plant macrofossil reconstructions in environmental governance has substantially increased [11,17,48]. In environmental governance, the idea of transforming lakes with turbid water conditions into a clear water environment is incorporated as a water reform program. In such programs, maintenance of the lake is usually accomplished by reducing algal density in the water column and minimizing human impacts [50]. The comparison of species occurrence and changing trends over the past century in Huanggai Lake and Liangzi Lake, located in the same climatic region, revealed that the information related to long-term historical macrophyte succession can guide lake restoration and protection programs efficiently in those lakes, which display similar ecological characteristics. Although both lakes shared the presence of similar dominant species prior to the severe human disturbance that began in the 1940s, the overall plant community structure changed significantly after the 1960s. Increased abundance of various macrophyte species—namely, *Potamogeton* sp., *Ceratophyllum demersum*, and *Myriophyllum spicatum*—preferring nutrient-rich lake environment (see Table 2), clearly indicates the gradual decline in a lacustrine environment over the recent decades and the need of better governance for restoration. However, in both of the lakes the presence of submerged species such as *Najas minor* and *Chara* sp., preferring clear water environments, prior to human intensifications in the 1940s, suggests that the success of environmental governance and restoration in the region lies in bringing the plant community dominated by *Najas minor* and *Chara* sp. to the shallow Yangtze lake system [48,55].

## 5. Conclusions

This study revealed that the reconstruction of aquatic vegetation succession in shallow Yangtze River lakes in China would significantly contribute to our knowledge of river restoration programs in the region. As the aquatic macrophytes are the key components of the shallow lake ecosystem for regulating climate and pollutants and purifying the water, macrofossils derived from Huanggai Lake sediment over the past century have clearly indicated different stages of plant species succession and lake evolution. A richer abundance of species such as *Najas minor* and *Vallisneria denseserrulata* in lake sediment prior to 1940 reflects the natural state of the lake ecosystem, with limited exposure to pollution, while the growth of emergent and floating plant species, *Potamogeton maackianus*, *Ceratophyllum demersum*, and *Potamogeton crispus* after 1974 suggests increasing anthropogenic impacts and vulnerability of shallow lake systems in the region. In the face of growing threats of climate change, population growth, and urbanization, a study of this kind is invaluable for shallow lake ecosystem management and water resources development around the middle and lower reaches of the Yangtze River Basin.

## Figures and Tables

**Table 1:** The main characteristics of water quality in Huanggai Lake.

Index	Summer 2004	Autumn 2007	Autumn 2011	Summer 2017
pH	9.14	8.62	8.05	8.90
TN (mg/L)	3.34	3.51	1.55	1.47
TP (mg/L)	0.14	0.167	0.113	0.093
Water quality grade ^1^	Ⅴ	Ⅴ	Ⅳ	Ⅳ

^1^ National Groundwater Quality Standard, People’s Republic of China (GB/T14848-9).

**Table 2:** Main aquatic plant groups and their ecological characteristics [31,37] in Huanggai Lake.

Family	Species	Life Span	Nutrient Requirement	Sensitivity to Environment
Najadaceae	*Najas minor* All.	Annual	Nutrient-poor	Sensitive
Hydrocharitaceae	*Vallisneria natans* (Lour.) Hara	Perennial	Meso-to-eutrophic	Medium tolerant
Hydrocharitaceae	*Vallisneria denseserrulata* (Makino) Makino	Perennial	Meso-to-eutrophic	Medium tolerant
Characeae	*Chara* sp.	Annual	Nutrient-poor	Sensitive
Hydrocharitaceae	*Hydrilla verticillata* (Linn. f.) Royle	Perennial	Meso-to-eutrophic	Nontolerant
Potamogetonaceae	*Potamogeton maackianus* A. Bennett	Perennial	Meso-to-eutrophic	Nontolerant
Potamogetonaceae	*Potamogeton crispus* L.	Perennial	Meso-to-eutrophic	Tolerant
Ceratophyllaceae Gray	*Ceratophyllum demersum* L.	Perennial	Meso-to-eutrophic	Tolerant
Haloragidaceae	*Myriophyllum spicatum* L.	Perennial	Middle-eutrophic	Tolerant

## Data Availability

Contact the correspondence author for data.
